# Generation of a 3D Liver Model Comprising Human Extracellular Matrix in an Alginate/Gelatin-Based Bioink by Extrusion Bioprinting for Infection and Transduction Studies

**DOI:** 10.3390/ijms19103129

**Published:** 2018-10-12

**Authors:** Thomas Hiller, Johanna Berg, Laura Elomaa, Viola Röhrs, Imran Ullah, Katrin Schaar, Ann-Christin Dietrich, Munir A. Al-Zeer, Andreas Kurtz, Andreas C. Hocke, Stefan Hippenstiel, Henry Fechner, Marie Weinhart, Jens Kurreck

**Affiliations:** 1 Institute of Biotechnology, Department of Applied Biochemistry, Technische Universität Berlin, 13355 Berlin, Germany; thomas.hiller@tu-berlin.de (T.H.); johanna.berg@tu-berlin.de (J.B.); viola.roehrs@tu-berlin.de (V.R.); katrin.schaar86@gmail.com (K.S.); a.dietrich@tu-berlin.de (A.-C.D.); al-zeer@tu-berlin.de (M.A.A.-Z.); henry.fechner@tu-berlin.de (H.F.); 2 Institute of Chemistry and Biochemistry, Department of Organic Chemistry, Freie Universität Berlin, 14195 Berlin, Germany; laura.elomaa@fu-berlin.de (L.E.); marie.weinhart@fu-berlin.de (M.W.); 3 Berlin-Brandenburger Centrum für Regenerative Therapien, Charité - Universitätsmedizin Berlin, 13353 Berlin, Germany; imran.ullah@charite.de (I.U.); Andreas.Kurtz@charite.de (A.K.); 4 Dept. of Internal Medicine/Infectious and Respiratory Diseases, Charité − Universitätsmedizin Berlin, 10117 Berlin, Germany; andreas.hocke@charite.de (A.C.H.); Stefan.Hippenstiel@charite.de (S.H.)

**Keywords:** adeno-associated virus, adenovirus, bioprinting, infection, transduction, extracellular matrix, liver, organ models, HepaRG, gene silencing

## Abstract

Bioprinting is a novel technology that may help to overcome limitations associated with two-dimensional (2D) cell cultures and animal experiments, as it allows the production of three-dimensional (3D) tissue models composed of human cells. The present study describes the optimization of a bioink composed of alginate, gelatin and human extracellular matrix (hECM) to print human HepaRG liver cells with a pneumatic extrusion printer. The resulting tissue model was tested for its suitability for the study of transduction by an adeno-associated virus (AAV) vector and infection with human adenovirus 5 (hAdV5). We found supplementation of the basic alginate/gelatin bioink with 0.5 and 1 mg/mL hECM provides desirable properties for the printing process, the stability of the printed constructs, and the viability and metabolic functions of the printed HepaRG cells. The tissue models were efficiently transduced by AAV vectors of serotype 6, which successfully silenced an endogenous target (cyclophilin B) by means of RNA interference. Furthermore, the printed 3D model supported efficient adenoviral replication making it suitable to study virus biology and develop new antiviral compounds. We consider the approach described here paradigmatic for the development of 3D tissue models for studies including viral vectors and infectious viruses.

## 1. Introduction

Three-dimensional (3D) bioprinting enables the fabrication of cell-laden biological 3D structures, which can contain multiple cell types as well as different biomaterials within a complex 3D geometry. Printed 3D constructs may provide suitable systems to study gene therapeutic strategies as well as tissue-specific infection of different pathogens. As they can be generated with human cells, they may reflect physiological conditions of humans better than animal experiments and thereby help to overcome shortcomings of current models. For instance, for a number of human pathogens, no appropriate animal model exists, as there are no natural hosts besides humans. In these cases, repeated passaging may succeed in adapting the pathogens to replicate in animal models; however, even when successful replication of the virus is achieved, the relevance of these models is often restricted and the pathophysiological manifestation of the infection is limited compared to their human equivalents [1,2,3]. For example, human adenovirus (AdV) does not replicate in mice and requires an elaborate model, e.g., with immunosuppressed Syrian hamsters, to study their biology [4]. AdV is a double-stranded DNA virus that usually causes mild, self-limiting infections of the upper respiratory tract and the gastrointestinal tract as well as epidemic conjunctivitis [5]. However, in immunocompromised patients following organ transplantation, AdV can cause severe infection with a fatal outcome [6]. Therapeutic options to treat severe courses of infection are limited, indicating the need to further study AdV biology and develop new antiviral agents.

Another important topic in current biomedical research is the development of efficient gene-delivery vehicles. Adeno-associated virus (AAV) vectors are among the most promising candidates for gene therapeutic applications [7] and have already been approved for clinical use [8,9]. Still, their toxicity and tropism of the different serotypes in human tissues needs to be characterized in more detail [10].

To overcome limits of currently established animal models, multicellular spheroids have been considered as in vitro 3D culture microscale tissue analogs; however, they could not be efficiently transduced with gene vectors [11,12]. Thus, 3D bioprinting offers an attractive alternative. Stereolithography printers generate models with high precision [13]. However, they require potentially harmful photo-inducible crosslinkers. Therefore, we chose an extrusion-based technology which allows biocompatible printing of various polymers such as collagen, gelatin, alginate, chitosan, silk or hyaluronic acid within a broad range of viscosities [14,15]. In particular, mixtures of alginate and gelatin combine the thermo-sensitive properties of gelatin [16,17,18] with the Ca^2+^-dependent cross-linking capabilities of alginate [19], thus providing properties well-suited for extrusion printing. Though these inks offer good printability, they lack desirable biochemical and physiological components. The addition of single extracellular matrix (ECM) components like collagen or laminin is a widely used strategy intended to get around this limitation, but they lack the diversity of natural ECM with respect to physical and biological complexity [20,21,22].

Thus, more complex human ECM (hECM) mimics like Matrigel™, which comprises a basement membrane protein composition similar to that found in human ECM [23,24], have been introduced for bioink optimization. However, Matrigel™, is extracted from a mouse tumor, the Engelbreth–Holm–Swarm sarcoma [25,26], which gives rise to ethical concerns. As a result, human ECM from decellularized human tissues is coming into focus for bioink optimization, since it provides a complex environment of essential human biomolecules. Despite its promising physiological properties, the application of ECM in 3D bioprinting approaches is difficult due to poor mechanical and viscoelastic properties [21,27] so that the composition of bioinks containing ECM needs to be optimized.

In addition to the bioink, the cells used in a bioprinting setup are of utmost importance. While primary cells, e.g., primary human hepatocytes (PHH) for a liver model, are the ultimate source of choice, their availability is limited and they are often highly susceptible to mechanical strain such as the shear stress during the printing process. Of all the currently available cell lines, the HepaRG cell line is the most appropriate alternative to PHH [28], since it shows comparable metabolic and morphologic properties. As a result, they are frequently applied for metabolic or cell biology studies [29,30,31].

The present study aims at optimizing conditions using human decellularized ECM as a component of an alginate/gelatin-based bioink, which can be used for extrusion-based bioprinting of a liver model for transduction and infection studies. Such a bioink must fulfil the requirements of printability, structural integrity (shape fidelity), and cell viability. Furthermore, it must support transduction by viral vectors and replication of infectious viruses. The hECM containing 3D constructs were generated using a 3D micro extrusion bioprinter. Initially, printing conditions, viability/cytotoxicity, and rheological properties were evaluated. In addition, the expression of metabolic markers over the course of the experiments was quantified. Transduction of the constructs with AAV vectors of serotype 6 was found to lead to efficient RNA interference (RNAi)-mediated silencing of the endogenous target human cyclophilin B (hCycB). Furthermore, the 3D constructs allowed proper replication of the human adenovirus 5 demonstrating their suitability for study of the virus.

## 2. Results and Discussion

### 2.1. Preparation and Characterization of HepaRG Cell-Laden Hydrogels Containing Varying Amounts of Extracellular Matrix (ECM)

**Figure 1:**
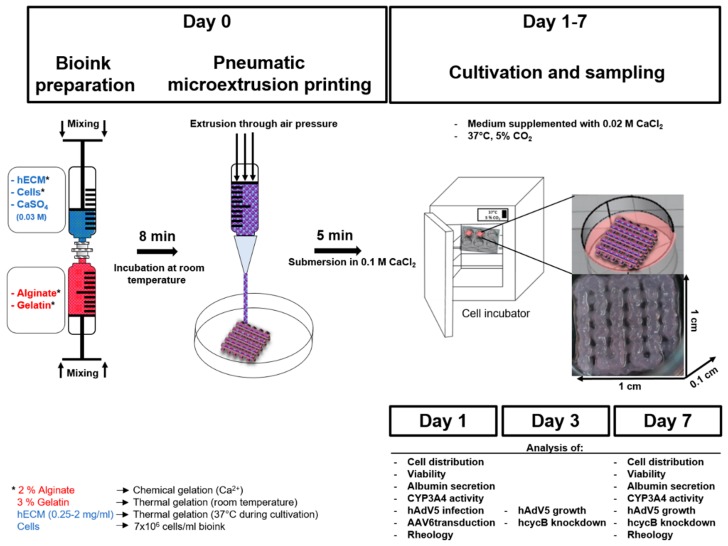
Experimental design of bioprinting and processing of cell-laden hybrid alginate/gelatin/human extracellular matrix (hECM) constructs. Schematic workflow of the 3D printing procedure. Bioink components and living mature HepaRG cells were thoroughly mixed with two syringes connected by a Luer-Lock-adapter. Following initial Ca^2+^-driven cross-linking, the hydrogel was transferred into the dispensing cartridge in the print head and pneumatically extruded onto a dry and sterile petri dish. The thermal gelation of gelatin maintained the 3D structure of the construct during the printing process. Alginate was completely cross-linked by submersion in a CaCl_2_ solution. During incubation at 37 °C, the gelatin dissolves, while the hECM gelation takes over support of the structural stability. Experiments were performed one, three and seven days after printing. * Detailed explanation of the function which the components of the bioink have.

The first aim of our study was to identify the optimal composition of a bioink to maintain HepaRG cultivation in a printed 3D construct. Although being of non-human origin, the suitability of alginate [19] and gelatin [16,17,18,32] for the fabrication of 3D scaffolds and bioprinting has been well established [33,34]. Our previous results for the 3D printing of lung epithelial cells showed favorable characteristics of blends containing alginate and gelatin in terms of printability, cell viability and preservation of the shape of the generated constructs during culture [35]. Therefore, the same basic bioink formulation consisting of 2% (*w*/*v*) alginate and 3% (*w*/*v*) gelatin was used to print mature HepaRG cells. This basic bioink was supplemented with varying amounts of human ECM. In the present study, we used lung-derived hECM for practical reasons. No substantial differences were reported for the composition of lung and liver ECM [36,37]. Therefore, we carried out this proof of concept study using lung-derived hECM in order to determine suitable hECM concentrations for extrusion-based bioprinting of HepaRG-laden hydrogels. Concentrations of 0, 0.25, 0.5, 1 and 2 mg/mL of decellularized hECM were added to the alginate/gelatin blend to determine the concentrations which are most beneficial in terms of improving biocompatibility and attachment of the cells to the matrix. The bioink contained 7 × 10^6^ mature HepaRG cells/mL, so that each construct should contain slightly less than 1 × 10^6^ HepaRG cells. In fact, when we lysed the constructs and counted the cells by trypan blue staining as previously described [35], we recovered approximately 750,000 cells from a model. We used a rectangular construct (length 1 cm × width 1 cm × height 0.1 cm) with regularly spaced pores laid out in a grid pattern for the experiments (Figure 1).

The bioinks used in the present study were of good printability and the shape of the constructs was highly reproducible (Supplementary Figure S1). Furthermore, the 3D models were stable during the cultivation time of seven days.

**Figure 2:**
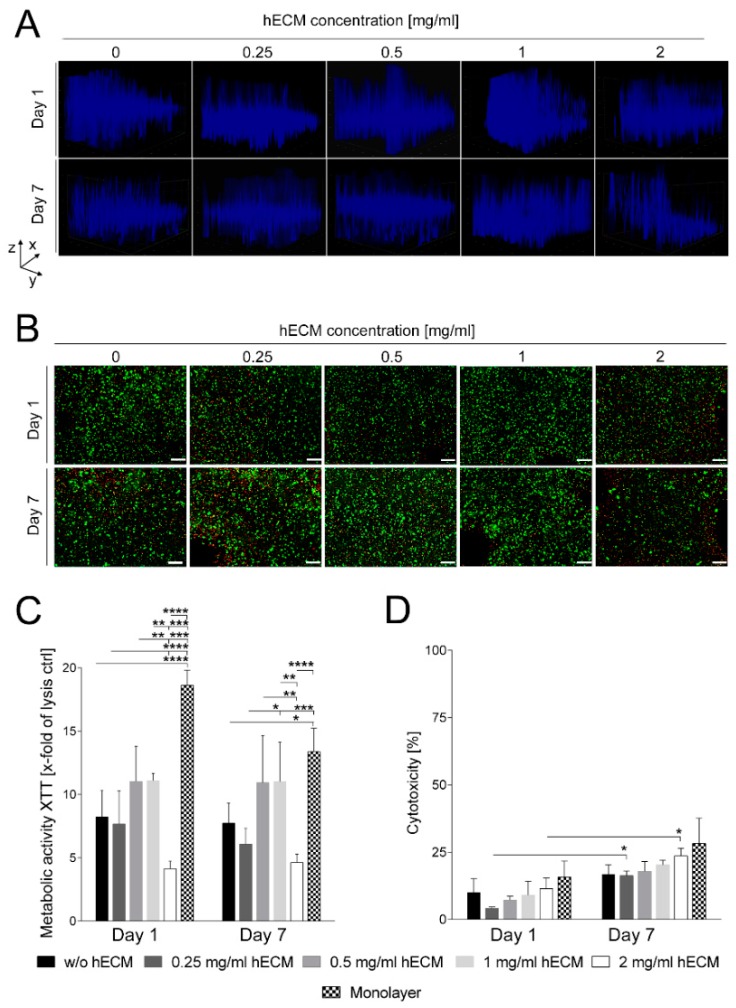
Spatial distribution and viability of mature HepaRG cells in 3D printed alginate/gelatin constructs with varying hECM concentrations. (**A**) Three-dimensional distribution of mature HepaRG cells in 3D printed constructs one and seven days after printing visualized by nuclear Hoechst staining (blue) and Z-stack analysis from the top of the gel to the dish surface (scanning depth 1000 µm, interval 15.12 µm, area 1800 × 1400 µm). 3D models were recorded with the Z-stack tool, which creates a projection of the transmitted light. Figure A shows representative images of the bioprinted 3D models carried out as three independent experiments; (**B**) Qualitative viability staining of living and dead mature HepaRG cells printed in the constructs after one and seven days of cultivation using calcein-AM (live in green) and ethidium homodimer-1 (dead in red). Scale bar: 200 µm; (**C**) Metabolic activity of mature HepaRG cells in different 3D printed alginate/gelatin/hECM bioinks was determined by the tetrazolium hydroxide salt (XTT) assay one and seven days after printing. Values were calculated as X-fold induction of lysis control; (**D**) Cytotoxicity was analyzed by the lactate dehydrogenase (LDH) assay. Data are depicted as percentage of the LDH level of cells in 3D printed constructs relative to the lysis control. For C and D, data from monolayer cultures are shown for comparison. Results are shown as mean ± standard error of the mean (SEM) of three independent experiments. * *p* ≤ 0.05; ** *p* ≤ 0.01; *** *p* ≤ 0.001; **** *p* ≤ 0.0001.

The spatial distribution of the mature HepaRG cells in the 3D printed constructs was analyzed by fluorescence microscopy. To visualize the cells, nuclei were stained with Hoechst stain and the 3D distribution was recorded with the Z-stack tool, which creates a projection of the transmitted light, following one and seven days in culture. Although this method is of limited resolution, it provides an overview over the cell distribution in the 3D printed construct. One day after printing, the HepaRG cells were well distributed throughout the printed constructs, with no obvious differences between the tested ECM concentrations (Figure 2A, upper row). At day seven after printing, the spatial distribution of the HepaRG cells was less homogenous and the cells tended to sediment in all constructs (Figure 2A, lower row); however, the bioink containing 1 mg/mL hECM was superior in maintaining spatial distribution of the cells compared to the other concentrations tested. Sections from the top, middle and bottom of the Z-stacks are shown in the Supplementary Figure S2.

Next, cell viability was qualitatively evaluated by staining living (calcein-AM, green) and dead (ethidium homodimer-1, red) cells after one and seven days of culture, followed by microscopic analysis. As obvious in Figure 2B (upper row), after one day of culture, cell viability was high in all bioink conditions except for 2 mg/mL hECM. This concentration resulted in a greater number of ethidium homodimer-1 positive, i.e., dead, cells compared to the other hECM concentrations. After seven days in culture (Figure 2B, lower row), the number of dead cells increased only slightly under all tested hECM conditions. Again, like after one day, the addition of 2 mg/mL hECM was detrimental as the percentage of dead cells was comparatively high. For constructs printed with bioinks containing 0.5 or 1 mg/mL hECM, the fraction of dead cells was also slightly lower than for those containing less hECM. While no differences in the number of calcein-AM positive, i.e., living, cells were detected after one day, seven days of culture with no or only 0.25 mg/mL hECM resulted in slightly reduced numbers of living HepaRG cells compared to 0.5 and 1 mg/mL hECM. Therefore, we concluded that hECM concentrations greater than 0.25 mg/mL and less than 2 mg/mL are best suited for cell viability.

The metabolic activity of the bioprinted mature HepaRG cells was determined by quantification of the reduction of the tetrazolium salt XTT by dehydrogenase enzymes after one and seven days in culture (Figure 2C). Consistent with the results from the microscopic evaluation of the cell staining, measurement of the metabolic activity of the bioprinted HepaRG revealed that 2 mg/mL hECM are unfavorable for cultivation of HepaRG cells, resulting in reduced metabolic activity levels. Even though slight differences regarding the enzymatic activity between the different hECM concentrations could be measured on days one and seven of culture, no significant decreases between day one and seven were detected at a given concentration of hECM.

The two-dimensional (2D) cultured mature HepaRG monolayer, which contained a comparable number of cells as the printed constructs, showed a significantly higher metabolic activity at day one (Figure 2C). However, metabolic activity in the monolayer culture decreased over time and was statistically no longer distinguishable on day seven of culture.

As an additional measure of metabolic activity, the release of lactate dehydrogenase (LDH) was measured to determine the cytotoxicity resulting from the different bioink conditions. Figure 2D shows that cytotoxicity of all tested bioink conditions was comparatively low (around 10% compared to the lysis control on day one after printing). A minor increase of about 5–10% was observed for the cultivation period of seven days, which is also typical for conventional 2D cell culture systems as also included in Figure 2D. Differences between day one and seven of culture were significant only for bioinks containing 0.25 and 2 mg/mL hECM.

The reduced viability of printed HepaRG cells at a concentration of 2 mg/mL hECM came as a surprise given the generally beneficial effects of ECM on cellular viability. The most abundant protein in the ECM is collagen [38], which is known to modulate the mechanical properties of tissues in vivo as well as in vitro dependent on its concentration [39,40]. One of the most common types of the 28 known collagens in mammals is type I collagen [41]. Collagen I monomers undergo fibrillar collagen formation at 37 °C and neutral pH values [42] to form hydrogels, a property which has been used in 3D bioprinting approaches [43,44,45,46]. The majority of the studies used low concentrations of collagen between 1 and 4 mg/mL. Likewise, most commercially available formulations contain low concentrations of collagen. In most of these studies, only a single concentration of collagen was used, leaving open the effects of varying concentrations on cellular behavior. Cross et al. showed that higher collagen concentrations (>20 mg/mL) restricted cell migration and viability of human vein endothelial cells due to the high density of the fibrillar structures [47]. This finding may explain our observation that the hECM concentration need to be high enough to support cell viability (≥0.5 mg/mL), but must not exceed a certain threshold of approximately 1 mg/mL to prevent detrimental effects. In addition, the stiffness of the construct should not exceed a certain level, which must still be determined, as this might negatively affect cell functionality.

### 2.2. Characterization of Hepatic Metabolism in HepaRG Cell-Laden Bioinks Containing Various Amounts of ECM

**Figure 3:**
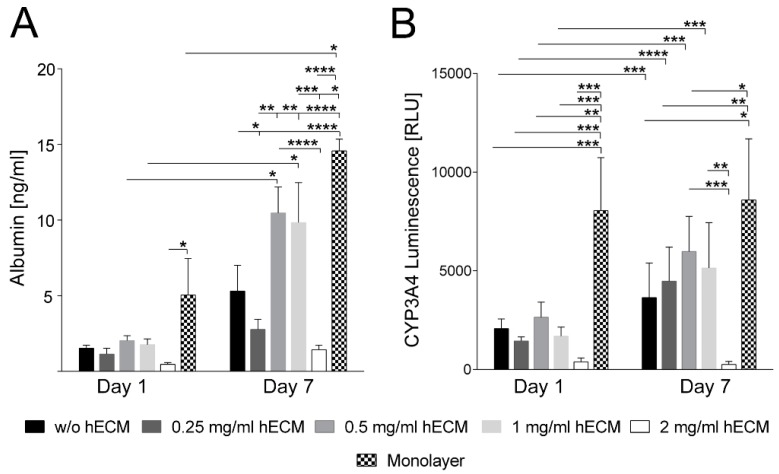
Albumin secretion and cytochrome P450 3A4 activity of printed mature HepaRG cells. (**A**) Quantitative enzyme-linked immunosorbent assay (ELISA) analysis of albumin secretion of mature HepaRG cell-laden 3D alginate/gelatin constructs with different ECM concentrations on day one and day seven; (**B**) CYP3A4 activity analysis of printed mature HepaRG cells determined by CYP3A4 induced luminescence. Comparison between the different ECM concentrations on day one and day seven. CYP3A4 luminescence was normalized to 10% Triton-X-100 treated cell lysis controls. Data from monolayer cultures are shown for comparison. Results are shown as mean ± SEM of three independent experiments. * *p* ≤ 0.05; ** *p* ≤ 0.01; *** *p* ≤ 0.001; **** *p* ≤ 0.0001.

To assess the impact of different hECM concentrations on the hepatic metabolism of printed HepaRG cells, albumin secretion and cytochrome P450 3A4 (CYP3A4) activity were analyzed. Albumin and CYP3A4 are two of the main markers for the characterization of hepatocytes. The production of albumin reflects the synthesis capacity of healthy cells and CYP3A4 their biotransformation activity [48,49]. HepaRG cells plated at low densities undergo morphological changes from an epithelial- and biliary-like phenotype to a hepatocyte-like phenotype [50,51]. The addition of dimethyl sulfoxide (DMSO) supports and maintains the hepatic maturation, which is accompanied by increased metabolic activities like albumin expression and cytochrome P450 activity [29,30,52]. One day after bioprinting of DMSO-treated mature HepaRG cells, no substantial differences in albumin secretion were observed between the different hECM concentrations tested (Figure 3A). The amount of secreted albumin then rose over time under all conditions tested; however, the increase was only statistically significant using 0.5 and 1 mg/mL hECM. At day seven of culture, the albumin level was approximately two- to threefold higher under these conditions compared to the other bioink compositions; the difference, again, being statistically highly significant.

CYP3A4 activity increased significantly from day one to day seven for all hECM concentrations except the highest, 2 mg/mL (Figure 3B). The differences between the CYP3A4 activity produced by the lower concentrations of hECM were not significant (Figure 3B). HepaRG cells cultured in the 2 mg/mL hECM-containing bioink showed significantly lower CYP3A4 activity on day seven compared to bioinks with other concentrations or no hECM (Figure 3B). In the absence of DMSO, the CYP3A4 activity is substantially lower (Supplementary Figure S3). Considering both albumin secretion and CYP3A4 activity, alginate/gelatin-based bioinks containing hECM concentrations of 0.5 or 1 mg/mL were found to be best-suited for mature HepaRG cell bioprinting. In our experiments bioinks with 0.5 or 1 mg/mL hECM triggered the highest HepaRG albumin secretion, as well as the highest CYP3A4 activity. In contrast, bioinks with 2 mg/mL hECM do not induce the tested hepatic activities, which may result from negative effects of high collagen concentrations on the metabolic conditions, as already discussed above for the LDH and XTT measurements.

Despite the elevated albumin secretion and CYP3A4 activity in 3D models printed with bioinks containing 0.5 and 1 mg/mL hECM, even higher metabolic activity was measured for HepaRG cells cultured in conventional 2D monolayers (Figure 3). A possible explanation is that the lower activity in 3D cultures is a result of the encapsulation of the cells in the bioink, as it is essential to safeguard the cells from pressure and shear stress occurring during the printing process [53]. This leaves the cells surrounded by a barrier-like layer of hydrogel, comparable to a sandwich culture, which has been associated with limitations in mass transport and drug sensitivity [54,55,56]. Consequently, it is plausible that secreted albumin and CYP3A4-formed luciferin were not completely released from the cells due to the surrounding hydrogel, or alternatively the diffusion of the CYP3A4 luminescence substrate into the hydrogel was insufficient or albumin release might have been incomplete due to interactions with the bioink material. Thus, the albumin and CYP3A4 luminescence values measured in the 3D culture may be underestimated compared to the ones in 2D cultured HepaRG cells, since a fraction of the measured metabolic parameters might not have been fully released from the hydrogel. In addition to limitations in mass transport, the encapsulation prevents cell–cell connection [35,44,57], which influences the measured metabolic activity of hepatocytes. For instance, hepatocytes, like HepaRG cells, cultured in spheroid models show increased metabolic activity compared to conventional monolayer culture [58,59]. Reasonable strategies to overcome this limitation and to further improve the bioinks presented here in this study have been published, e.g., the use of spheroids instead of single cell suspensions [60] or modified hydrogels that are sensitive to matrix metalloproteases (MMPs) which can be used to degrade the cell encapsulation [57]. Another approach to optimize the bioink is the inclusion of liver-derived, instead of lung-derived hECM, as it might contain non-collagenous proteins that may influence liver-specific metabolism.

### 2.3. Characterization of Rheological Properties of ECM-Based Hydrogels

**Figure 4:**
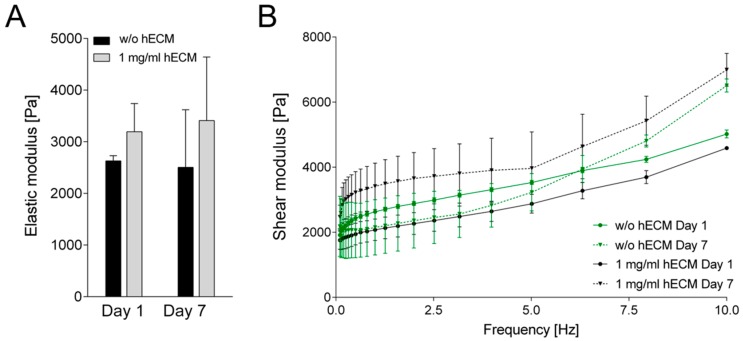
Rheological properties of 3D printed alginate/gelatin constructs with varying hECM concentration. (**A**) Comparison between 0 and 1 mg/mL hECM, one and seven days after printing. Elastic modulus of the wet bioink formulations were measured at a frequency of 1 Hz and 0.1% shear strain at 37 °C; (**B**) Shear modulus of 3D printed constructs at increasing frequencies (0.1–10 Hz). Results are shown as mean ± SEM of a triplicate experiment.

The initial experiments have shown that the addition of 0.5–1 mg/mL hECM was well-suited with respect to cell viability and metabolic activity. Furthermore, experimental handling and accurate maintenance of the printed structure were improved at higher hECM concentrations so that all further experiments were carried out with bioinks containing 1 mg/mL hECM. The next step was to investigate rheological features of the hydrogels. Elastic properties of the printed constructs were measured three and seven days after printing using an oscillating rheometer at a frequency sweep of 0.1–10 Hz at 0.1% shear strain. No significant changes in the elastic modulus were observed between days one and seven of the experiment, either in the presence or in the absence of hECM (Figure 4). The printed constructs without hECM generally showed a slightly lower elastic modulus compared to the hECM-containing ones on both days of measurement. Taken together, the rheological analysis confirmed that the 3D printed hydrogels with or without hECM did not lose their mechanical integrity within the time frame of the experiments. It should be noted that the hECM used in the present study originates from a single donor. Possible donor-to-donor variability will have to be tested in future studies.

### 2.4. Transduction of Bioprinted Liver Model with Adeno-Associated Virus (AAV) Vectors

**Figure 5:**
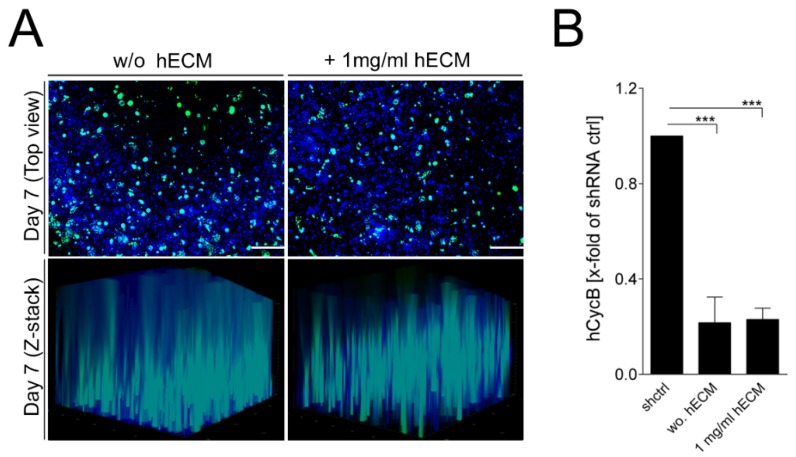
Adeno-associated virus (AAV) transduction and hCycB silencing in printed mature HepaRG cells in alginate/gelatin bioinks containing 1 mg/mL hECM. (**A**) Analysis of AAV2.6 vector transduction and distribution within mature HepaRG cell-laden 3D alginate/gelatin constructs, determined by fluorescence microscopy. Comparison of constructs with and without 1 mg/mL hECM seven days after printing. Nuclei were visualized by Hoechst staining (blue); the green fluorescence represents green fluorescent protein (GFP) expression of AAV vectors. Scale bar: 200 µm; (**B**) Analysis of shRNA-mediated hCycB RNA knockdown within the mature HepaRG cell-laden 3D alginate/gelatin constructs, determined by reverse transcription polymerase chain reaction (RT-qPCR). Comparison of constructs with and without 1 mg/mL hECM seven days after transduction. An shRNA control was used for normalization to assess the knockdown. Results are shown as mean ± SEM of three independent experiments. *** *p* ≤ 0.001.

AAV vectors are efficient tools for gene delivery without inducing any recognized pathogenicity [7,61,62,63,64]. They are particularly well-suited for application in RNAi approaches [65]. Pseudotyped AAV2.6 vectors (AAV2.6) display a pronounced liver tropism [66], and so they were used in the transduction experiments. Mature HepaRG-laden constructs cultured with or without 1 mg/mL hECM were transduced with 1 × 10^5^ AAV2.6/cell for seven days. Independent of the concentration of hECM used, high AAV vector transduction rates were observed, as well as an even spatial distribution within the printed 3D constructs seven days post-transduction. The ability of the AAV2.6 vectors to transduce mature HepaRG cells was characterized by observing the expression of the encoded marker EmGFP, which was analyzed by fluorescence microscopy. The overview images in Figure 5A demonstrate widespread transduction of the cells in the printed constructs. Sections from the top, middle and bottom of the Z-stacks are shown in the Supplementary Figure S4.

Furthermore, the functional applicability of the approach was determined by an RNAi experiment as the vectors also expressed a small hairpin RNA (shRNA) directed against hCycB, an endogenously expressed target that is well-suited to investigate silencing efficiency [67,68]. In the printed 3D tissue models, transduction with AAV2.6 mediated an average knockdown of hCycB of 70–80% compared to the shRNA control, which was used for normalization to assess the knockdown efficiency (Figure 5B). As no differences were observed for the bioinks containing no or 1 mg/mL hECM, we conclude that the addition of hECM is not detrimental to the transduction efficiency of the AAV vectors. Furthermore, the printed model and geometry including pores support widespread transduction of all cells. In a recent publication, we estimated by scanning electron microscopy that the pore size of an optimized alginate/gelatin bioink supplemented with Matrigel is about 1–2 µm [35]. It is reasonable to assume that the alginate/gelatin bioink with hECM used here has a comparable pore size. This can explain the efficient penetration by AAV vectors we observed, since they are two orders of magnitude smaller. This contrasts with bulky spheroids that are often too dense for large particles like viral vectors to penetrate the 3D culture right to the center.

### 2.5. Adenovirus Infection of Bioprinted Liver Model

**Figure 6:**
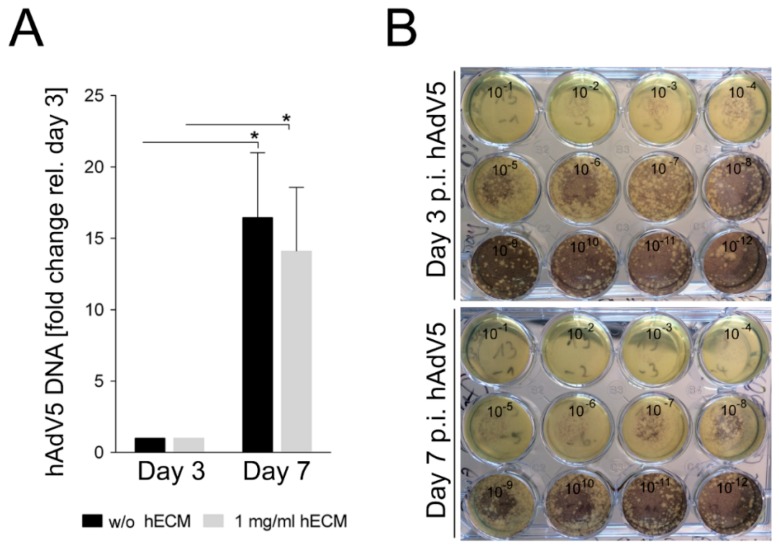
Adenovirus infection of printed mature HepaRG cells in alginate/gelatin bioinks containing hECM. (**A**) Analysis of adenoviral DNA replication, within the mature HepaRG cell-laden 3D alginate/gelatin constructs, determined by quantitative polymerase chain reaction (qPCR). Comparison of constructs without and with 1 mg/mL hECM three and seven days after infection. The adenoviral DNA amount three days post infection with Adv5 was used as reference to assess the course of viral infection seven days post infection. Results are shown as mean ± SEM of three independent experiments. * *p* ≤ 0.05; (**B**) Cell-killing assay: A549 cells were infected with serial dilutions of the hAdV5 containing supernatants of the infected 3D constructs and subsequently covered with low melting agar. Plaques were stained with 2-MTT-INT staining solution seven days after infection.

To further analyze whether the hECM-containing 3D-printed models allow viral infection and replication, they were infected with hAdV5 according to the scheme published in [4]. In an analogy to testing of transduction efficiency, constructs containing no hECM or 1 mg/mL hECM were infected with a multiplicity of infection (MOI) of 10 and incubated for three or seven days. The relative amount of adenoviral DNA was quantified by quantitative polymerase chain reaction (qPCR) analysis of the level of the adenoviral hexon gene and the housekeeping 18S rRNA. The adenoviral DNA amount three days post infection with hAdv5 was used as baseline to assess the yield of viral DNA seven days post infection. An approximately increase in the concentration of the adenoviral hexon DNA occurred between days three and seven post-infection. The addition of hECM did not have a statistically significant influence, indicating efficient replication of hAdV5 in the 3D-printed mature HepaRG-laden constructs (Figure 6A). Since the presence of adenoviral DNA does not directly reflect the production of infectious viral particles, we infected A549 cells with serial dilutions of the hAdV5-containing supernatants from the infected 3D cultures. An increase of infectious adenoviral particles was obvious between days three and seven post-hAdV5 infection (Figure 6B). Samples taken three days post hAdV5 infection efficiently lysed A549 cell monolayers down to a dilution of 10^−6^. In contrast, the samples taken seven days post-hAdV5 infection even resulted in lysed A549 monolayers down to dilutions between 10^−8^ and 10^−9^. This result correlates nicely with the increase of adenoviral hexon DNA in the supernatants of the hAdV5 infected 3D constructs and also indicates an increase of the number of infectious particles in the models, independent of the presence of hECM. The printed model is thus suitable to study virus biology in a humanized 3D cell culture, and the addition of 1 mg/mL hECM is not detrimental to AdV5 replication.

## 3. Materials and Methods

### 3.1. Cell Culture and Human Extracellular Matrix Preparation

Human bipotent hepatic progenitor cells (HepaRG; Biopredic, Saint Gregoiré, France) were cultured in William’s E medium without l-glutamine (Gibco, Dreieich, Germany) supplemented with 10% fetal bovine serum (FBS; c.c.pro, Oberdorla, Germany), 2 mM l-Glutamine (Biowest, Nuaillé, France), 5 μg/mL recombinant human insulin (PAN Biotech, Aidenbach, Germany), 50 µM hydrocortisone hemisuccinate (Sigma, Steinheim, Germany), and 1% penicillin/streptomycin (P/S; Biowest). After 14 days, hepatic maturation was induced for additional 14 days by application of 1.7% DMSO (Sigma) to the culture medium.

Human epithelial lung carcinoma cells (A549; ATCC, Manassas, VA, USA) were cultured using Dulbecco´s Modified Eagle Medium (DMEM) high glucose (Biowest) supplemented with 10% fetal bovine serum (FBS; c.c.pro), 2 mM l-Glutamine (Biowest) and 1% penicillin/streptomycin (P/S; Biowest).

Human extracellular matrix (hECM) was isolated from a lung of a deceased patient. The lung was obtained from the German Heart Center Berlin and the study was approved by the ethics committee at the Charité clinic (project: EA2/079/13). For decellularization, lung pieces were subjected to 0.1% sodium dodecyl sulfate at room temperature for 4 h, incubated in 350 IU/mL DNase1 for two hours (room temperature) and sterilized in phosphate-buffered saline (PBS) supplemented with 100 U/mL penicillin and 100 µg/mL streptomycin for 2 h. After lyophilization, hECM powder was dissolved in a pepsin solution of 1 mg/mL (pH 2.0; prepared in 0.01 M HCl) to obtain a final hECM concentration of 10 mg/mL. The digested hECM solution was then neutralized with 10× PBS (pH 7.4) and 0.1 M NaOH (for a detailed protocol, supplementary information). The final protein concentration was measured with the Pierce BCA-200 Protein Assay Kit (Thermo Fisher Scientific, Dreieich, Germany), according to the manufacturer’s instructions, at an absorbance of A620 nm (Sunrise absorbance microplate reader, Tecan, Männedorf, Switzerland).

### 3.2. Preparation of Cell-Laden Biopolymers

Gelatin (6.7% *w*/*v*) and sodium alginate (4.5% *w*/*v*) powder (Sigma) were dissolved in William’s E medium with supplements on a magnetic stirrer at 1250 min^−1^, at 37 °C overnight. The hybrid gelatin/alginate hydrogel was then mixed with hECM in various concentrations, mature HepaRG cells, CaSO_4_ (Roth, Karlsruhe, Germany) and William’s E medium with supplements. The final cell-laden bioink was composed of 2% *w*/*v* alginate, 3% *w*/*v* gelatin, 0; 0.25; 0.5; 1, or 2 mg/mL *w*/*v* hECM as indicated, 0.03 M CaSO_4_, and 7 × 10^6^ mature HepaRG cells/mL. Following CaSO_4_-driven initial cross-linking of alginate (8 min after mixing), the cell-laden bioink was loaded into the printing cartridge.

### 3.3. 3D Bioprinting

For the bioprinting process, the bioink was extruded through a 22G needle at 10–20 kPa with the microextrusion printer INKREDIBLE+ (Cellink, Gothenburg, Sweden). The 3D construct was designed by the computer-aided design (CAD) software Rhinoceros5 (Robert McNeel & Associates, Barcelona, Spain). The printed constructs were submerged in 0.1 M CaCl_2_ (Roth) to increase gelation of alginate and subsequently cultured in an incubator at 37 °C and 5% CO_2_ in William’s E medium with supplements and 1.7% DMSO, as well as 0.02 M CaCl_2_ for up to seven days.

### 3.4. Rheological Properties

The viscoelastic behavior of the 3D printed constructs was analyzed using a Kinexus lab+ oscillating rheometer (Malvern, Malvern, UK) with an active hood Peltier plate cartridge (Malvern). Before testing, samples were maintained for pre-determined time at 37 °C and 5% CO_2_ in William’s E medium with supplements, representing the cell culture conditions. An elastic modulus of the wet 3D printed hydrogels was recorded within a viscoelastic regime by running a frequency sweep of 0.1–10 Hz at 0.1% shear strain. The measurements were run at 37 °C using an 8 mm parallel plate geometry.

### 3.5. Cell Distribution

Printed cell-laden 3D constructs were fixed in 4% formaldehyde (Sigma), permeabilized with 1% Triton-X-100 (Roth) for 15 min and the nuclei were stained with 1 µg/mL Hoechst stain (H33342, AppliChem, Darmstadt, Germany) for 1 h at room temperature. Cellular distribution was analyzed with the Zeiss Observer. Z1 microscope (Zeiss, Jena, Germany).

### 3.6. Cell Viability and Lactate Dehydrogenase Release

Metabolic activity of mature HepaRG cells was determined using the XTT assay according to the manufacturer’s instructions (AppliChem). Briefly, XTT reagent (1 mg/mL) was added on cell-laden 3D constructs and incubated for 4 h (37 °C, 5% CO_2_). The absorbance of the supernatant was measured spectrophotometrically at A450 nm (TriStar Multimode Reader LB942, Berthold Technologies, Bad Wildbad, Germany) with a reference of A620 nm.

Lactate dehydrogenase (LDH) release of mature HepaRG cells was measured with the LDH detection kit (Roche, Grenzach, Germany), according to the manufacturer’s instructions, in the supernatant at an absorbance of A492 nm with a reference of A620 nm (Sunrise absorbance microplate reader, Tecan). XTT and LDH values were normalized to lysis controls. For cell lysis, cell-laden 3D constructs were incubated in culture medium supplemented with 10% Triton-X-100.

The LIVE/DEAD assay of mature HepaRG cells was performed with the Viability/Cytotoxicity kit (Thermo Fisher Scientific), according to the manufacturer’s instructions. Briefly, cell-laden 3D constructs were stained with 2 µM calcein-AM and 2 µM ethidium homodimer-1 diluted in 1x Hank’s balanced salt solution (HBSS; Thermo Fisher Scientific) for 30 min (37 °C, 5% CO_2_). The stained cell-laden 3D constructs were analyzed by fluorescence microscopy (Zeiss Observer. Z1 microscope).

### 3.7. Albumin Secretion and Cytochrome P450 3A4 Activity

Secreted albumin was quantified in the culture supernatant with the Human Albumin Enzyme-Linked Immunosorbent Assay (ELISA) Kit (Bethyl laboratories, Montgomery, TX, USA), according to the manufacturer’s instructions, at an absorbance of A450 nm (TriStar Multimode Reader LB942) with a reference of A620 nm.

Cytochrome P450 oxidase 3A4 (CYP3A4) activity was determined with the P450-Glo CYP3A4 Assay (Promega, Mannheim, Germany), according to the manufacturer’s instructions. Briefly, CYP3A4 substrate Luciferin-PFBE (50 µM) was added on cell-laden 3D constructs and incubated at 37 °C, 5% CO_2_. After 4 h, CYP3A4-mediated conversion of Luciferin-PFBE substrate to luciferin, which is secreted from the printed HepaRG cells, was determined. The supernatant was incubated with Luciferin detection reagent and the luminescence was measured (TriStar Multimode Reader LB942). Luminescence values were normalized to lysis controls. For cell lysis, cell-laden 3D constructs were incubated in culture medium supplemented with 10% Triton-X-100.

### 3.8. Adeno-Associated Virus Production, Transduction, and hCycB Knockdown

Pseudotyped scAAV vectors of serotype 6 (AAV2.6) encoding an expression cassette for an shRNA against hCycB and Emerald green fluorescent protein (EmGFP), were produced, purified and titrated as previously described [69]. HepaRG-laden 3D constructs were transduced with 1 × 10^5^ AAV/cell in William’s E medium with supplements without DMSO. To determine transduction efficiency, EmGFP-expression in transduced HepaRG cells was analyzed by fluorescence microscopy (Zeiss Observer. Z1 microscope) seven days after transduction. To quantify hCycB knockdown, RNA for RT-qPCR experiments (see below) was isolated with the NucleoSpin TriPrep (MACHEREY NAGEL, Düren, Germany) according to manufacturer’s instructions.

### 3.9. Human Adenovirus 5 Preparation and Infection

Human adenovirus 5 (hAdV5), obtained from Stefan Weger (Institute of Virology, Campus Benjamin Franklin, Charité-Universitätsmedizin, Berlin, Germany), was grown on HEK293 cells. Viral titers were determined by standard plaque assay on HEK293 cells [4]. For adenoviral infection, cell-laden 3D printed constructs were washed three times with 1× HBSS and inoculated with hAdV5 (MOI of 10) for 30 min at room temperature. Afterwards, William’s E medium without l-glutamine supplemented with 2% FBS, 2 mM l-Glutamine, 5 μg/mL recombinant human insulin, 50 µM hydrocortisone hemisuccinate, and 1% P/S without DMSO was added, and the cell-laden 3D constructs were incubated at 37 °C and 5% CO_2_. Twenty-four hours after the printing procedure, the 3D tissues were infected with AdV5. Three and seven days post infection, the adenovirus-containing supernatants were collected for adenoviral replication analysis by qPCR experiments (see below).

### 3.10. Cell-Killing Assay

Cell-laden 3D constructs were infected and incubated as described above. On days 3 and 7 after infection, the adenovirus-enriched supernatant was collected, diluted (10^−1^–10^−12^) in DMEM high glucose supplemented with 2% FBS, 1% 100× l-Glutamine, 1% P/S, and transferred on A549 cells. After reinfection overnight, the cells were covered with 5% low melting point agar (Sigma) in DMEM high glucose supplemented with 5% FBS, 1% 100× l-Glutamine, 1% P/S and incubated for 24 h (37 °C; 5% CO_2_). After seven days, the covered cells were stained with 2 × 3-(4,5-Dimethylthiazol-2-yl)-2,5-diphenyltetrazolium bromide (MTT)-2-(4-Iodophenyl)-3-(4-nitrophenyl)-5-phenyltetrazolium chloride (VWR-Chemicals, Dresden, Germany) for 2 h (37 °C; 5% CO_2_) to determine the viral effect.

### 3.11. Reverse Transcription and Quantitative Polymerase Chain Reaction (qPCR)

The expression of hCycB was quantified by RT-qPCR as described previously [69]. Briefly, equal amounts of RNA were subjected to reverse transcription (RT) using the RevertAid H Minus First Strand cDNA Synthesis Kit and random hexamer primers (MBI Fermentas, St. Leon-Rot, Germany) according to the manufacturer’s instructions.

For qPCR of hCycB and 18S rRNA cDNAs and adenoviral hexon DNA, the SsoFastEvaGreen Supermix (BioRad, München, Germany) was used. Reaction mixtures, thermocycling programs and primer sequences are described in Supplementary Tables S1–S3. The relative hCycB RNA expression was normalized to 18S rRNA and determined by the ΔΔ*C*_t_ method. Adenoviral hexon DNA was evaluated by the Δ*C*_t_ method.

### 3.12. Statistical Analysis

Statistical evaluation of experiments was performed using two-way analysis of variance (ANOVA) with Bonferroni correction (GraphPad Prism 6, GraphPad Software, Inc.; La Jolla, CA, USA). Each set of cell-laden experiments was repeated in minimum of three times. Data are represented as mean ± standard error of the mean (SEM), *p* values are considered significant by * *p* ≤ 0.05; ** *p* ≤ 0.01; *** *p* ≤ 0.001, **** *p* ≤ 0,0001.

## 4. Conclusions

Taken together, our findings demonstrate that supplementation of an alginate/gelatin bioink with 0.5–1 mg/mL hECM improves cell viability and hepatic metabolic activity (Figure 2C and Figure 3) in a humanized 3D liver model generated by extrusion bioprinting. The bioink provides high precision in the printing process and stability of the printed constructs during prolonged cultivation. Supplementary Figure S5 shows some additional 3D shapes that were printable with the bioink. The hECM was not detrimental to transduction of the tissue model by AAV vectors or its infection with human adenovirus 5. It improved the properties of the bioink for the bioprinting process and for the accurate maintenance of a defined 3D structure. ECM from human donors can thus be used to replace supporting protein mixtures such as Matrigel™ that are widely used, but have to be harvested from mouse tumors. The use of human ECM thus reduces the number of animals needed to obtain the supporting material for bioinks and avoids problems that may arise from species-specific differences between mouse and human. In addition, the hECM used is not of tumorous origin and provides a normal environment for the cells. Future studies should look into the possibility that liver-derived hECM behaves differently than lung-derived and attempt to replace the components of non-human origin in the bioink by materials found in the human organism. In addition, cellular interaction with the matrix may be further improved by the addition of proteinaceous components such as RGD (Arg-Gly-Asp) motifs. The design of the tissue model printed here allowed widespread transduction of the cells in the construct, which is not possible in large spheroids which tend to be too dense for penetration by large particles such as viruses and viral vectors to the center. Therefore, we conclude that extrusion-based bioprinting of 3D tissue models with a bioink containing human ECM is a suitable approach to generate models for transduction and transfection studies.

## Supplementary Materials

Supplementary materials can be found at http://www.mdpi.com/1422-0067/19/10/3129/s1.

## Abbreviations

AAV	Adeno-associated virus
AAV2.6	Pseudotyped scAAV vector of serotype 6
AdV	Adenovirus
hAdV5	Human adenovirus serotype 5
MOI	Multiplicity of infection
ECM	Extracellular matrix
hECM	Human extracellular matrix
hCycB	Human cyclophillin b
qPCR	Quantitative polymerase chain reaction
RNAi	RNA interferencelinear dichroism
shRNA	Small hairpin RNA
EmGFP	Emerald green fluorescent protein
CYP3A4	Cytochrome P450 oxidase 3A4

## References

[1] Guha C., Mohan S., Roy-Chowdhury N., Roy-Chowdhury J. (2004). Cell culture and animal models of viral hepatitis. Part I: Hepatitis B. Lab. Anim. (NY).

[2] Hough R., Chetwood A., Sinfield R., Welch J., Vora A. (2005). Fatal adenovirus hepatitis during standard chemotherapy for childhood acute lymphoblastic leukemia. J. Pediatr. Hematol. Oncol..

[3] Bouvier N.M., Lowen A.C. (2010). Animal Models for Influenza Virus Pathogenesis and Transmission. Viruses.

[4] Schaar K., Geisler A., Kraus M., Pinkert S., Pryshliak M., Spencer J.F., Tollefson A.E., Ying B., Kurreck J., Wold W.S. (2017). Anti-adenoviral Artificial MicroRNAs Expressed from AAV9 Vectors Inhibit Human Adenovirus Infection in Immunosuppressed Syrian Hamsters. Mol. Ther. Nucleic Acids.

[5] Berk A.J., Knipe D.M., Howley P.M. (2007). Adenoviridae: The viruses and Their Replication. Fields Virology.

[6] Baldwin A., Kingman H., Darville M., Foot A.B., Grier D., Cornish J.M., Goulden N., Oakhill A., Pamphilon D.H., Steward C.G. (2000). Outcome and clinical course of 100 patients with adenovirus infection following bone marrow transplantation. Bone Marrow Transplant..

[7] Kay M.A. (2011). State-of-the-art gene-based therapies: The road ahead. Nat. Rev. Genet..

[8] (2018). Voretigene neparvovec-rzyl (Luxturna) for inherited retinal dystrophy. Med. Lett. Drugs Ther..

[9] Yla-Herttuala S. (2010). Endgame: Glybera finally recommended for approval as the first gene therapy drug in the European union. Mol. Ther..

[10] Lisowski L., Tay S.S., Alexander I.E. (2015). Adeno-associated virus serotypes for gene therapeutics. Curr. Opin. Pharmacol..

[11] Enger P.O., Thorsen F., Lonning P.E., Bjerkvig R., Hoover F. (2002). Adeno-associated viral vectors penetrate human solid tumor tissue in vivo more effectively than adenoviral vectors. Hum. Gene Ther..

[12] Thorsen F., Afione S., Huszthy P.C., Tysnes B.B., Svendsen A., Bjerkvig R., Kotin R.M., Lonning P.E., Hoover F. (2006). Adeno-associated virus (AAV) serotypes 2, 4 and 5 display similar transduction profiles and penetrate solid tumor tissue in models of human glioma. J. Gene Med..

[13] Grix T., Ruppelt A., Thomas A., Amler A.K., Noichl B.P., Lauster R., Kloke L. (2018). Bioprinting Perfusion-Enabled Liver Equivalents for Advanced Organ-on-a-Chip Applications. Genes (Basel).

[14] Ozbolat I.T., Peng W., Ozbolat V. (2016). Application areas of 3D bioprinting. Drug Discov. Today.

[15] You F., Eames B.F., Chen X. (2017). Application of Extrusion-Based Hydrogel Bioprinting for Cartilage Tissue Engineering. Int. J. Mol. Sci..

[16] Wang X., Yan Y., Pan Y., Xiong Z., Liu H., Cheng J., Liu F., Lin F., Wu R., Zhang R. (2006). Generation of three-dimensional hepatocyte/gelatin structures with rapid prototyping system. Tissue Eng..

[17] Wang X., Xu H. (2010). Incorporation of DMSO and dextran-40 into a gelatin/alginate hydrogel for controlled assembled cell cryopreservation. Cryobiology.

[18] Billiet T., Gevaert E., De Schryver T., Cornelissen M., Dubruel P. (2014). The 3D printing of gelatin methacrylamide cell-laden tissue-engineered constructs with high cell viability. Biomaterials.

[19] Axpe E., Oyen M.L. (2016). Applications of Alginate-Based Bioinks in 3D Bioprinting. Int. J. Mol. Sci..

[20] Hospodiuk M., Dey M., Sosnoski D., Ozbolat I.T. (2017). The bioink: A comprehensive review on bioprintable materials. Biotechnol. Adv..

[21] Choudhury D., Tun H.W., Wang T., Naing M.W. (2018). Organ-Derived Decellularized Extracellular Matrix: A Game Changer for Bioink Manufacturing?. Trends Biotechnol..

[22] Lee H., Han W., Kim H., Ha D.H., Jang J., Kim B.S., Cho D.W. (2017). Development of Liver Decellularized Extracellular Matrix Bioink for Three-Dimensional Cell Printing-Based Liver Tissue Engineering. Biomacromolecules.

[23] Kleinman H.K., McGarvey M.L., Liotta L.A., Robey P.G., Tryggvason K., Martin G.R. (1982). Isolation and characterization of type IV procollagen, laminin, and heparan sulfate proteoglycan from the EHS sarcoma. Biochemistry.

[24] Hughes C.S., Postovit L.M., Lajoie G.A. (2010). Matrigel: A complex protein mixture required for optimal growth of cell culture. Proteomics.

[25] Kleinman H.K., Martin G.R. (2005). Matrigel: Basement membrane matrix with biological activity. Semin. Cancer Biol..

[26] Benton G., Arnaoutova I., George J., Kleinman H.K., Koblinski J. (2014). Matrigel: From discovery and ECM mimicry to assays and models for cancer research. Adv. Drug Deliv. Rev..

[27] Eo J.S., Soo H.J., Jong-Ock S., Kyung-Hwan J., Nam-Soo K. (2017). Extracellular Matrix and 3D Printing. Curr. Trends Biomed. Eng. Biosci..

[28] Nibourg G.A., Chamuleau R.A., van Gulik T.M., Hoekstra R. (2012). Proliferative human cell sources applied as biocomponent in bioartificial livers: A review. Expert Opin. Biol. Ther..

[29] Aninat C., Piton A., Glaise D., Le Charpentier T., Langouet S., Morel F., Guguen-Guillouzo C., Guillouzo A. (2006). Expression of cytochromes P450, conjugating enzymes and nuclear receptors in human hepatoma HepaRG cells. Drug Metab. Dispos..

[30] Kanebratt K.P., Andersson T.B. (2008). Evaluation of HepaRG cells as an in vitro model for human drug metabolism studies. Drug Metab. Dispos..

[31] Marion M.J., Hantz O., Durantel D. (2010). The HepaRG cell line: Biological properties and relevance as a tool for cell biology, drug metabolism, and virology studies. Methods Mol. Biol..

[32] Rahali K., Ben Messaoud G., Kahn C.J.F., Sanchez-Gonzalez L., Kaci M., Cleymand F., Fleutot S., Linder M., Desobry S., Arab-Tehrany E. (2017). Synthesis and Characterization of Nanofunctionalized Gelatin Methacrylate Hydrogels. Int. J. Mol. Sci..

[33] You F., Wu X., Chen D.X.B. (2016). 3D printing of porous alginate/gelatin hydrogel scaffolds and their mechanical property characterization. Int. J. Pol. Mater. Pol. Biomater..

[34] Lou Y., Li Y., Qin X., Wa Q. (2018). 3D printing of concentrated alginate/gelatin scaffolds with homogeneous nano apatite coating for bone tissue engineering. Mater. Des..

[35] Berg J., Hiller T., Kissner M.S., Qazi T.H., Duda G.N., Hocke A.C., Hippenstiel S., Elomaa L., Weinhart M., Fahrenson C. (2018). Optimization of cell-laden bioinks for 3D bioprinting and efficient infection with influenza A virus. Sci. Rep..

[36] Denner J., Tonjes R.R. (2012). Infection barriers to successful xenotransplantation focusing on porcine endogenous retroviruses. Clin. Microbiol. Rev..

[37] Beachley V.Z., Wolf M.T., Sadtler K., Manda S.S., Jacobs H., Blatchley M.R., Bader J.S., Pandey A., Pardoll D., Elisseeff J.H. (2015). Tissue matrix arrays for high-throughput screening and systems analysis of cell function. Nat. Methods.

[38] Gelse K., Poschl E., Aigner T. (2003). Collagens—Structure, function, and biosynthesis. Adv. Drug Deliv. Rev..

[39] Miron-Mendoza M., Seemann J., Grinnell F. (2010). The differential regulation of cell motile activity through matrix stiffness and porosity in three dimensional collagen matrices. Biomaterials.

[40] Antoine E.E., Vlachos P.P., Rylander M.N. (2014). Review of collagen I hydrogels for bioengineered tissue microenvironments: Characterization of mechanics, structure, and transport. Tissue Eng. Part. B Rev..

[41] Ricard-Blum S. (2011). The collagen family. Cold Spring Harb. Perspect. Biol..

[42] Williams S.K., Hoying J.B., Turkse K. (2015). Bioprinting in Regenrative Medicine.

[43] Roth E.A., Xu T., Das M., Gregory C., Hickman J.J., Boland T. (2004). Inkjet printing for high-throughput cell patterning. Biomaterials.

[44] Wu Z.J., Su X., Xu Y.Y., Kong B., Sun W., Mi S.L. (2016). Bioprinting three-dimensional cell-laden tissue constructs with controllable degradation. Sci. Rep..

[45] Lee V., Singh G., Trasatti J.P., Bjornsson C., Xu X., Tran T.N., Yoo S.S., Dai G., Karande P. (2014). Design and fabrication of human skin by three-dimensional bioprinting. Tissue Eng. Part. C Methods.

[46] Park J.Y., Choi J.C., Shim J.H., Lee J.S., Park H., Kim S.W., Doh J., Cho D.W. (2014). A comparative study on collagen type I and hyaluronic acid dependent cell behavior for osteochondral tissue bioprinting. Biofabrication.

[47] Cross V.L., Zheng Y., Won Choi N., Verbridge S.S., Sutermaster B.A., Bonassar L.J., Fischbach C., Stroock A.D. (2010). Dense type I collagen matrices that support cellular remodeling and microfabrication for studies of tumor angiogenesis and vasculogenesis in vitro. Biomaterials.

[48] Basheer L., Kerem Z. (2015). Interactions between CYP3A4 and Dietary Polyphenols. Oxid. Med. Cell. Longev..

[49] Raoufinia R., Mota A., Keyhanvar N., Safari F., Shamekhi S., Abdolalizadeh J. (2016). Overview of Albumin and Its Purification Methods. Adv. Pharm. Bull..

[50] Gripon P., Rumin S., Urban S., Le Seyec J., Glaise D., Cannie I., Guyomard C., Lucas J., Trepo C., Guguen-Guillouzo C. (2002). Infection of a human hepatoma cell line by hepatitis B virus. Proc. Natl. Acad. Sci. USA.

[51] Parent R., Marion M.J., Furio L., Trepo C., Petit M.A. (2004). Origin and characterization of a human bipotent liver progenitor cell line. Gastroenterology.

[52] Hoekstra R., Nibourg G.A., van der Hoeven T.V., Ackermans M.T., Hakvoort T.B., van Gulik T.M., Lamers W.H., Elferink R.P., Chamuleau R.A. (2011). The HepaRG cell line is suitable for bioartificial liver application. Int. J. Biochem. Cell. Biol..

[53] Gasperini L., Mano J.F., Reis R.L. (2014). Natural polymers for the microencapsulation of cells. J. R. Soc. Interface.

[54] Xu J.J., Henstock P.V., Dunn M.C., Smith A.R., Chabot J.R., de Graaf D. (2008). Cellular imaging predictions of clinical drug-induced liver injury. Toxicol. Sci..

[55] Kimoto E., Walsky R., Zhang H., Bi Y.A., Whalen K.M., Yang Y.S., Linder C., Xiao Y., Iseki K., Fenner K.S. (2012). Differential modulation of cytochrome P450 activity and the effect of 1-aminobenzotriazole on hepatic transport in sandwich-cultured human hepatocytes. Drug Metab. Dispos..

[56] Lauschke V.M., Hendriks D.F., Bell C.C., Andersson T.B., Ingelman-Sundberg M. (2016). Novel 3D Culture Systems for Studies of Human Liver Function and Assessments of the Hepatotoxicity of Drugs and Drug Candidates. Chem. Res. Toxicol..

[57] Sun J., Wei D., Yang K., Liu X., Hongsong F., Zhang X. (2017). The development of cell-initiated degradable hydrogel based on methacrylated alginate applicable to multiple microfabrication technologies. J. Mater. Chem. B.

[58] Gunness P., Mueller D., Shevchenko V., Heinzle E., Ingelman-Sundberg M., Noor F. (2013). 3D organotypic cultures of human HepaRG cells: A tool for in vitro toxicity studies. Toxicol. Sci..

[59] Takahashi Y., Hori Y., Yamamoto T., Urashima T., Ohara Y., Tanaka H. (2015). 3D spheroid cultures improve the metabolic gene expression profiles of HepaRG cells. Biosci. Rep..

[60] Mironov V., Visconti R.P., Kasyanov V., Forgacs G., Drake C.J., Markwald R.R. (2009). Organ printing: Tissue spheroids as building blocks. Biomaterials.

[61] Fechner H., Pinkert S., Geisler A., Poller W., Kurreck J. (2011). Pharmacological and biological antiviral therapeutics for cardiac coxsackievirus infections. Molecules.

[62] Kay M.A., Nakai H. (2003). Looking into the safety of AAV vectors. Nature.

[63] Santiago-Ortiz J.L., Schaffer D.V. (2016). Adeno-associated virus (AAV) vectors in cancer gene therapy. J. Control. Release.

[64] Grimm D., Kay M.A. (2003). From virus evolution to vector revolution: Use of naturally occurring serotypes of adeno-associated virus (AAV) as novel vectors for human gene therapy. Curr. Gene Ther..

[65] Grimm D., Kay M.A. (2007). RNAi and Gene Therapy: A Mutual Attraction. Hematol. Am. Soc. Hematol. Educ. Program..

[66] Jiang H., Lillicrap D., Patarroyo-White S., Liu T., Qian X., Scallan C.D., Powell S., Keller T., McMurray M., Labelle A. (2006). Multiyear therapeutic benefit of AAV serotypes 2, 6, and 8 delivering factor VIII to hemophilia A mice and dogs. Blood.

[67] Hiller T., Rohrs V., Dehne E.M., Wagner A., Fechner H., Lauster R., Kurreck J. (2016). Study of Viral Vectors in a Three-dimensional Liver Model Repopulated with the Human Hepatocellular Carcinoma Cell Line HepG2. J. Vis. Exp..

[68] Wagner A., Rohrs V., Materne E.M., Hiller T., Kedzierski R., Fechner H., Lauster R., Kurreck J. (2015). Use of a three-dimensional humanized liver model for the study of viral gene vectors. J. Biotechnol..

[69] Wagner A., Rohrs V., Kedzierski R., Fechner H., Kurreck J. (2013). A novel method for the quantification of adeno-associated virus vectors for RNA interference applications using quantitative polymerase chain reaction and purified genomic adeno-associated virus DNA as a standard. Hum. Gene Ther. Methods.

