# Supplementary material for: Generation of a 3D Liver Model Comprising Human Extracellular Matrix in an Alginate/Gelatin-Based Bioink by Extrusion Bioprinting for Infection and Transduction Studies
Source: Int J Mol Sci. 2018 Oct 12;19(10):3129. doi: 10.3390/ijms19103129 (PMC6213460; doi:10.3390/ijms19103129)
Supplement: Supplementary file 1 [file ijms-19-03129-s001.pdf]

Supplement

Methods

Human extracellular matrix preparation

For decellularization, the lung was cut into approximately 1 cm^3 cuboid pieces and subsequently sectioned into 600 µm thick slices using a cryostat. Every slice was transferred into one well of a 6 well plate and washed with distilled water for 120 min at 4°C (water was changed every 30 min). After washing, the slices were subjected to 0.1% sodium dodecyl sulfate (SDS) at room temperature for 4 h (SDS solution was changed every 30 min). For SDS removal, slices were washed with distilled water for 30 min at 4°C (water was changed every 10 min). After washing, the slices were incubated in 350 IU/mL DNase1 for 2 h at room temperature (DNase1 solution was changed every hour). For DNase1 removal, slices were washed with distilled water for 30 min at 4°C (water was changed every 10 min). For sterilization, decellularized tissue slices were washed in PBS supplemented with 100 U/ml penicillin and 100µg/ml streptomycin for 2 h (PBS was changed every 30 min).

After decellularization, slices were lyophilized and the resulting hECM powder was dissolved in a porcine pepsin solution of 1 mg/mL (pH 2.0; prepared in 0,01 M HCl) to obtain a final hECM concentration of 10 mg/ml. After two days with continuous shaking at 27° C, the digested hECM solution was neutralized with 10x PBS (pH7.4) and 0.1 M NaOH (example for 10 ml hECM solution: mix 6.9 ml hECM solution with 2.1 ml cold 10x PBS (pH 7.4) and 1 ml cold 0.1 M NaOH) in a precooled tube on ice.

Quantitative PCR

Supplementary Table S1: Reaction mixture used for quantitative polymerase chain reaction.

Component	Final concentration
Mastermix	1x
Forward primer	250 nM
Reverse primer	250 nM
1 µl of template DNA or cDNA was used in a 20 µl reaction mix	

(according to *Wagner et al. 2013*).

Supplementary Table S2: Thermocycling programs used for quantitative polymerase chain reaction.

	Target	Target	
	18s and adenoviral hexon	*hCycB**	
Step 1	95 °C / 30 sec	95 °C / 30 sec	
Step 2	95 °C / 5 sec	95 °C / 5 sec	40 cycles
Step 3	64 °C / 5 sec	60 °C / 5 sec	
Melting curve	65- 95 °C , increment 0.5 °C		

(*according to *Wagner et al. 2013*).

Supplementary Table S3: Primer and sequences used for quantitative polymerase chain reaction.

Primer	Sequence (5´to 3´)
18 s RNA forward*	CGCGGTTCTATTTTGTTGGT
18 s RNA reverse*	AGTCGGCATCGTTTATGGTC
hCycB forward*	CTGCGCCTCTCCGAACGCAA
hCycB reverse*	CATCGGCCGCAGAAGGTCCC
Adenoviral hexon forward**	CACATCCAGGTGCCTCAGAA
Adenoviral hexon reverse**	AGGTGGCGTAAAGGCAAATG

(*according to *Wagner et al. 2013;* **according to Schaar *et al.* 2017).

Results and discussion

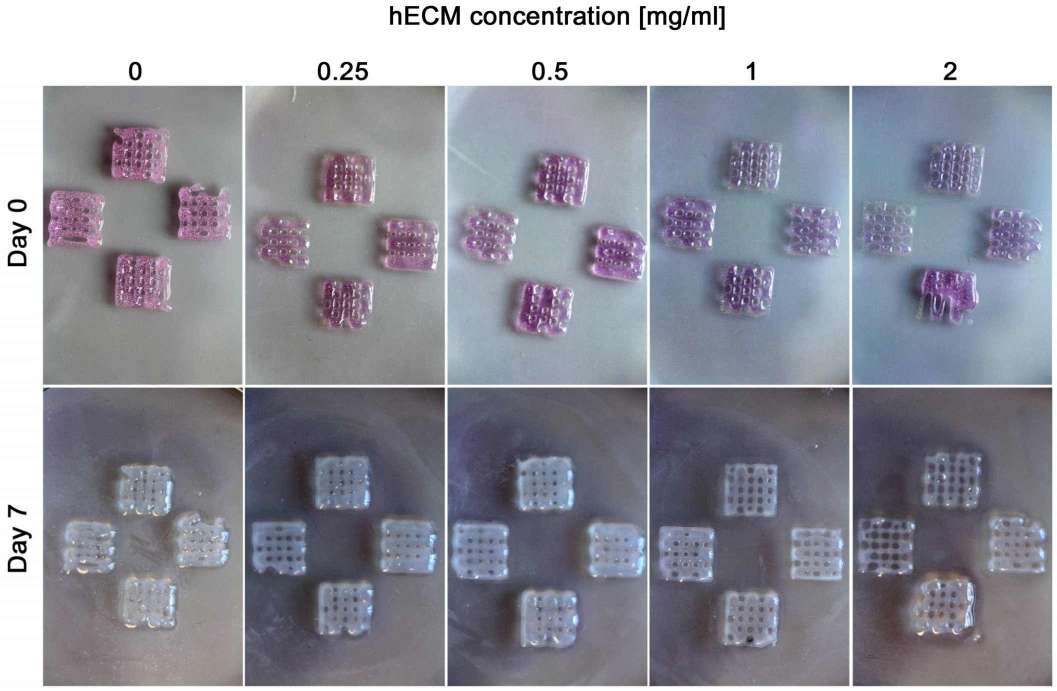

Supplementary Figure S1. Reproducibility of the printing process and stability of the printed constructs. Bioinks contained the basic alginate/gelatin composition and the indicated concentrations of hECM. Cells were not included for the integrity assays. To demonstrate reproducibility four constructs were printed for each condition. Furthermore, stability of the constructs after seven days is shown.

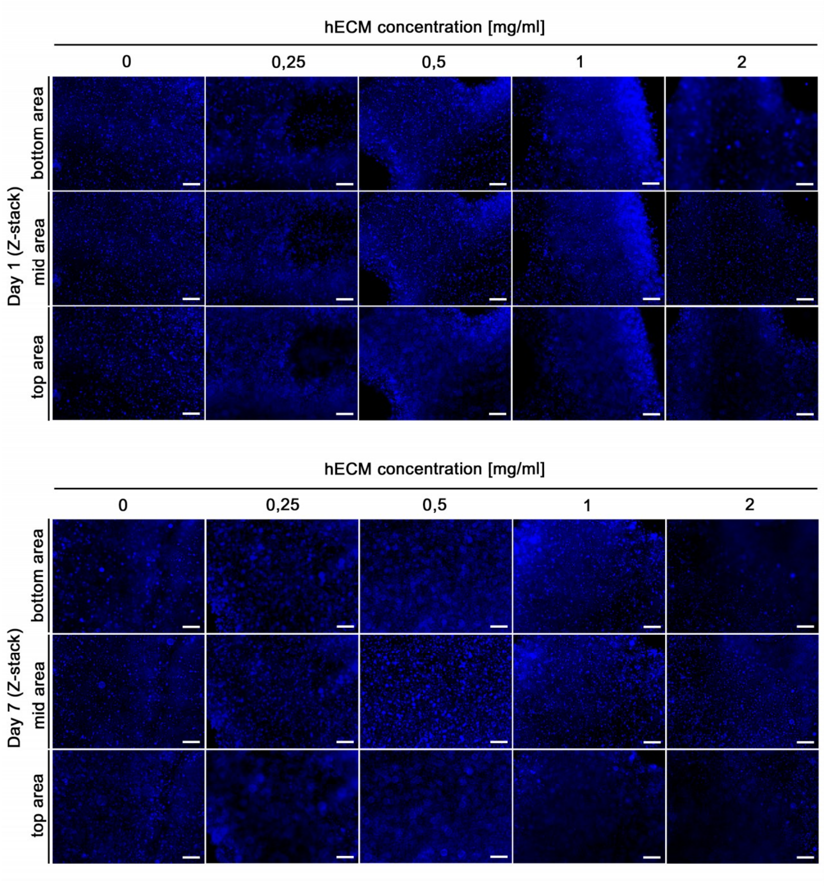

Supplementary Figure S2. Sections of the Z-stacks shown in Figure 2. The figure shows sections from the top, middle and bottom of the Z-stack of constructs printed with varying hECM concentrations, on days one and seven of cultivation. Scale bar: 200 μm.

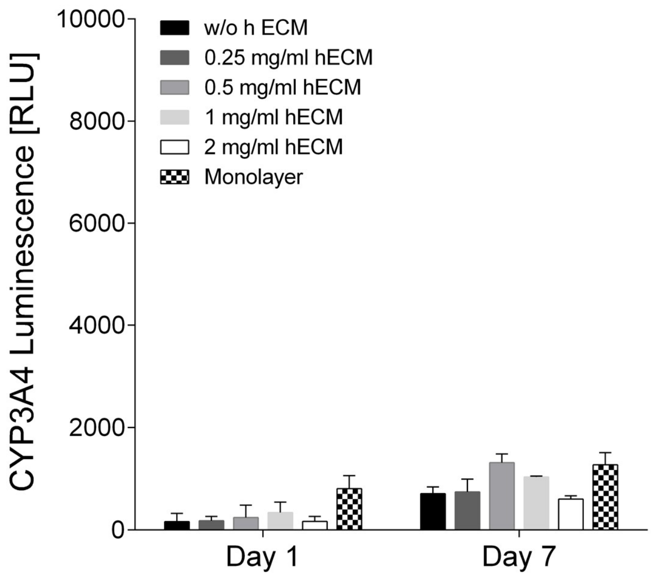

Supplementary Figure S3. CYP3A4 activity of HepaRG cells cultivated in the absence of DMSO. CYP3A4 activity was measured on day 1 and 7 in constructs containing different amounts of hECM and in monolayer culture. CYP3A4 luminescence was normalized to 10% Triton-X-100 treated cell lysis controls.

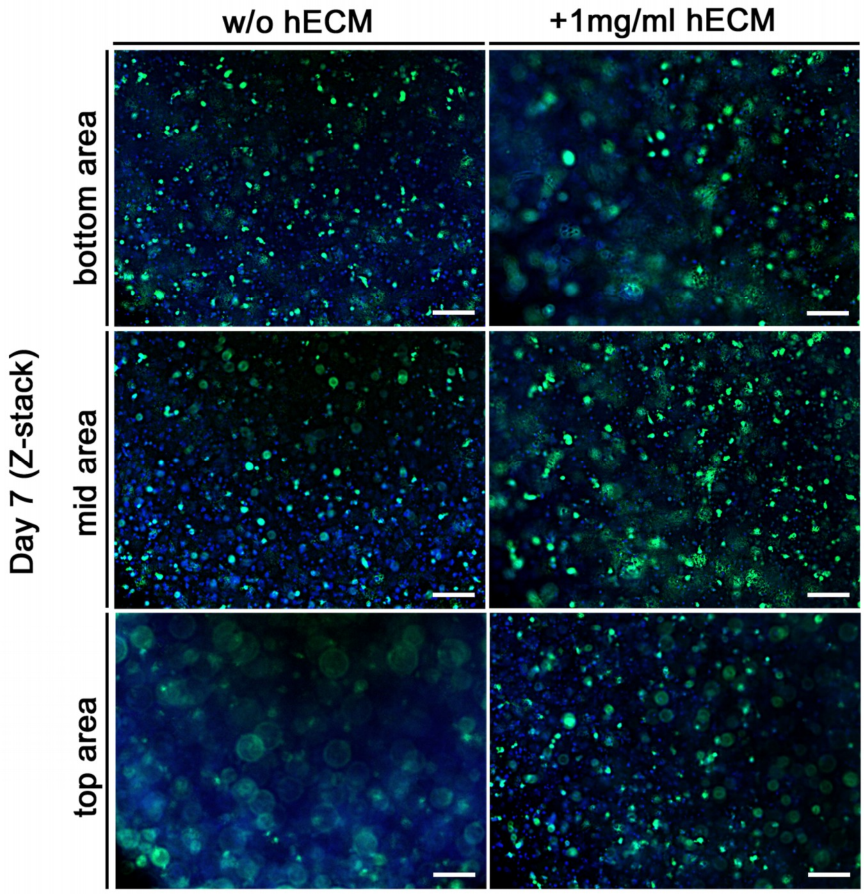

Supplementary Figure S4. Sections of the Z-stacks shown in Figure 5. The figure shows sections from the top, middle and bottom of the Z-stack of constructs transduced with AAV2.6 vectors, on day seven after transduction. Scale bar: 200 µm.

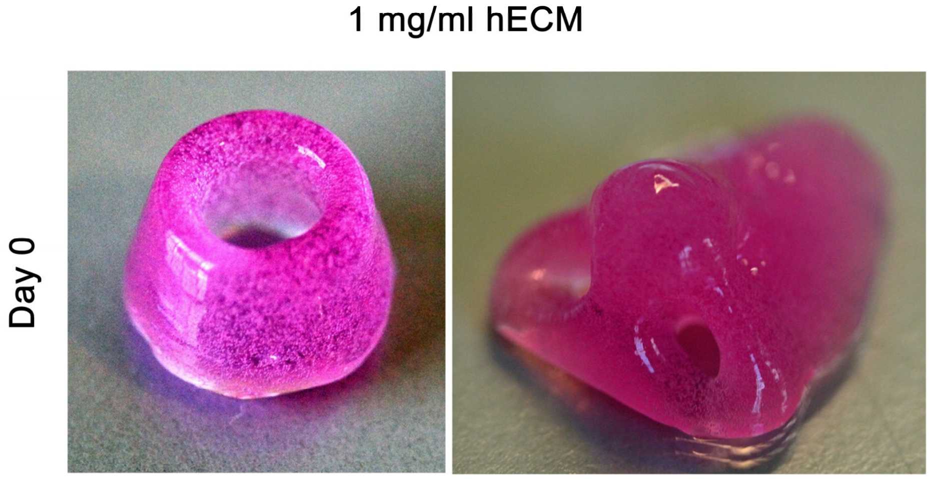

Supplementary Figure S5: Additional shapes printed with the optimized bioink composed of alginate, gelatine and 1 mg/ml hECM.
